# Effect of 50 Hz electric field in diacylglycerol acyltransferase mRNA expression level and plasma concentration of triacylglycerol, free fatty acid, phospholipid and total cholesterol

**DOI:** 10.1186/1476-511X-11-68

**Published:** 2012-06-07

**Authors:** Takuya Hori, Shinji Harakawa, Shirley M Herbas, Yoshiko Y Ueta, Noboru Inoue, Hiroshi Suzuki

**Affiliations:** 1 Hakuju Institute for Health Science, Tokyo, 151-0063, Japan; 2 National Research Center for Protozoan Diseases, Obihiro University of Agriculture and Veterinary Medicine, Inada, Obihiro, 080-8555, Japan

**Keywords:** Diacylglycerol O-acyltransferase, Electric field

## Abstract

**Background:**

The effects of exposure to a 50 Hz electric field (EF) on plasma level of triacylglycerol, free fatty acids, total cholesterol and phospholipid and mRNA expression level of diacylglycerol acyltransferase (DGAT) 1 and 2 in liver and intestines from C57BL/6 J mice were studied.

**Methods:**

The test was based on comparison between mice post treated with 50 Hz EF of 45 kV/m intensity for 30 min per day for 11 days or without EF. DGATs mRNA expression was analyzed by real-time quantitative polymerase chain reaction.

**Results:**

There was no difference in the gene expression level of DGAT1 in liver and intestines. The DGAT2 gene expression level in liver derived from mice treated with EF was significantly lower than those in the control (P < 0.001). Both plasma total cholesterol (P < 0.01) and phospholipid (P < 0.05) in the group exposed to EF were lower than those in the control, but there was no difference in triacylglycerol or free fatty acid levels.

**Conclusion:**

Exposure to 50 Hz EF decrease the plasma levels of total cholesterol and phospholipids, and downregulated DGAT2 mRNA expression in liver. The mechanisms for the effects of EF on lipid metabolism are not well understand yet, but altered DGAT2 activity may be involved.

## Background

Recently, electricity usage has increased and is widespread in both household and industry. Most important fields of research regarding this is the investigation of the possible biological effects of power-line frequency, 50 or 60 Hz electric fields (EF) [1-4]. Another major line of research concerning the beneficial efficacies of EFs involves the use of electrical devices e.g., treating bone diseases and stimulating growth [5,6]. These devices, which were first approved for use by the US Food Drug Administration in 1978, involve the application of electrical energy to the treated region, resulting in induced electric currents that mediate the desired therapeutic effect. In 1972, the Ministry of Health and Welfare, Tokyo, Japan approved the manufacture of an alternating current high voltage electric field health device, an apparatus that utilizes 50 or 60 Hz EF without generating magnetic field, and supposedly a physical therapeutic instrument in the alleviation of pain related to shoulder stiffness, insomnia, chronic constipation, and headaches [7].

Our previous report suggests suppressive effects of 50 Hz EF on place avoidance response [8], and that the exposure of C57BL/6 J male mice to EF improves their subfertility activity in mating with superovulated females [9]. In addition, our results also indicated that EF depressed plasma adrenocorticotropic hormone levels due to an immobilization [10]. The findings referred to above were obtained with the same EF conditions. Although we have also reported some findings regarding the mechanisms of our observed EF-mediated changes [11], our findings and those of other groups still remain largely unexplained. For instance, our previous study on the effects of EF on lipid metabolism demonstrates that triacylglycerol or free fatty acid levels in plasma is reduced by a 50- or 60-Hz sinusoidal EF [12,13]. Recently, Coskun et al., using identical EF conditions, also reported a similar phenomenon in rat [14].

Herein, we quantified the expression of diacylglycerol O-acyltransferase (DGAT) genes, which encode essential enzymes involved in the synthesis of triacylglycerol. We also measured blood properties including triacylglycerol, free fatty acids, total cholesterol and phospholipids which are correlated with the lipogenesis pathway.

## Results

### Analysis of DGAT mRNA expression in the liver and intestines by real-time quantitative polymerase chain reaction

**Figure 1:**
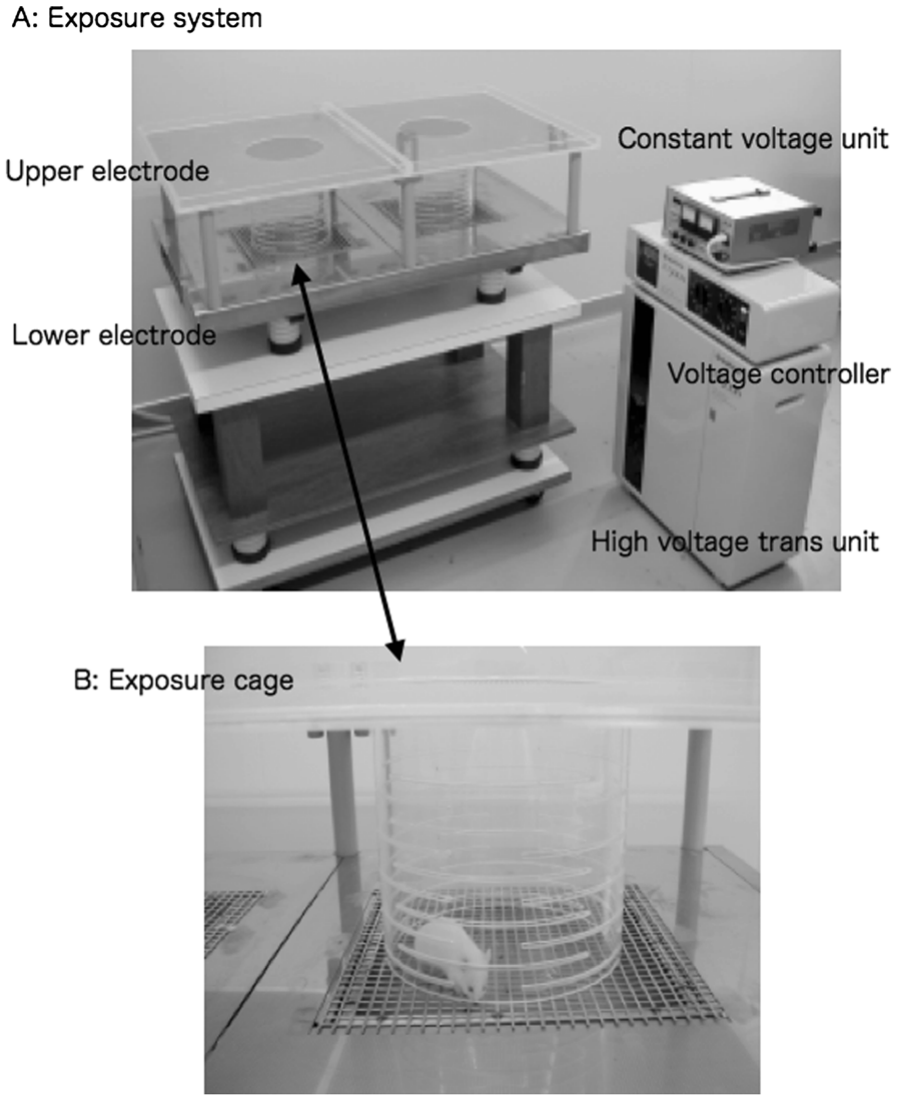
**Quantitative assessments of DGAT1 (A) and DGAT2 (B), mRNA expression levels were analyzed by real-time quantitative PCR (n = 6)**. The means of the 2 groups with respect to DGAT2 are statistically different (P < 0.001).

DGAT 1 and 2 mRNA expression levels in liver and small and large intestine tissues are shown in Figure 1A and B. DGAT1 mRNA expression of small intestine was higher compared with that of other tissues, but no differences were not observed between the EF and sham groups. The expression level of DGAT2 mRNA in liver was higher than that in intestines and the values from livers of the EF-exposed group were significantly lower than that in the control group (P < 0.001).

### Analysis of blood triacylglycerol, free fatty acids, total cholesterol and phospholipids

**Figure 2:**
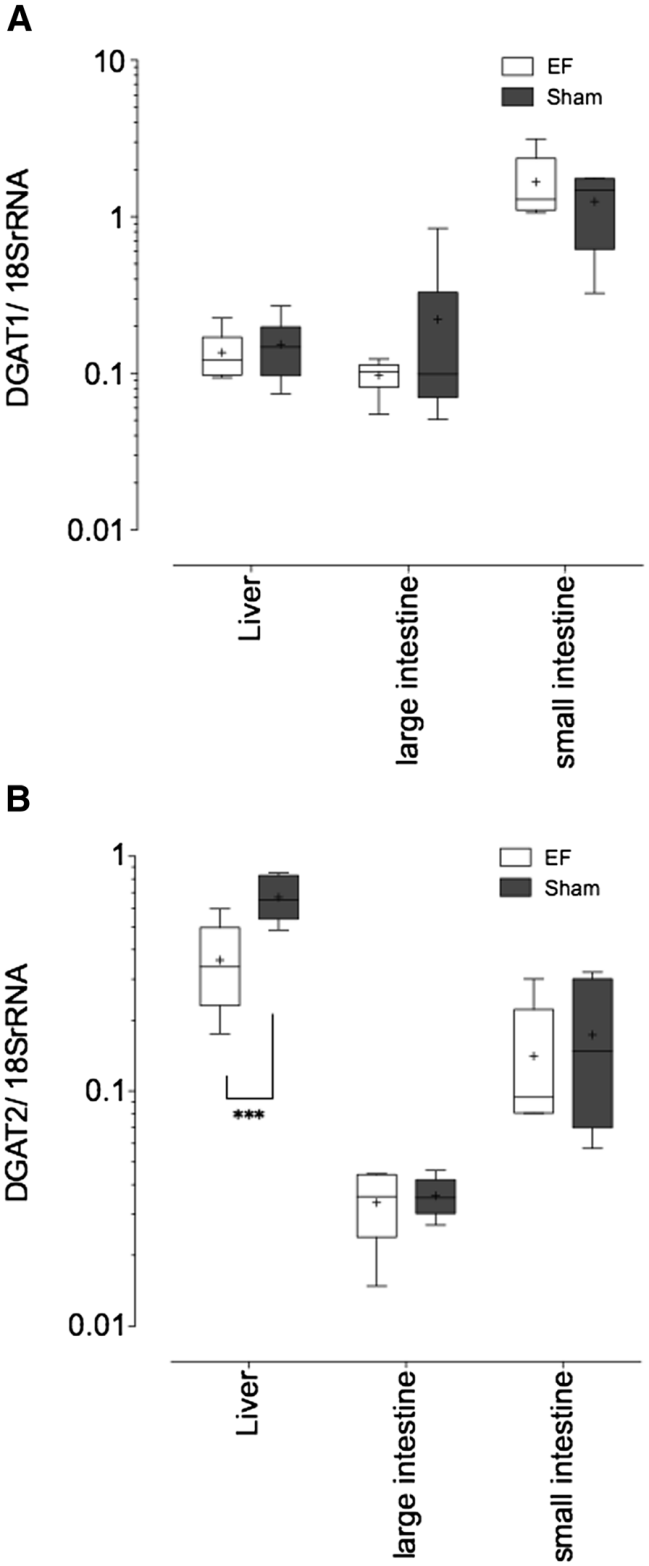
** Plasma phospholipid in the EF treatment group was significantly lower than that in the sham group (P < 0.05)**. Total cholesterol in the EF treatment group was also significantly lower than that in the sham group (P < 0.01).

Plasma phospholipid levels in the EF-exposed group were lower than that in the control group (P < 0.05, Figure 2). Levels of total plasma cholesterol in the EF-exposed group exposed were also lower than that in the control group (P < 0.01, Figure 2). The levels of triacylglycerol and free fatty acids were not different between groups.

## Discussion

DGAT is a microsomal enzyme that catalyzes the final and only committed step in the glycerol phosphate pathway [15-18]. The gene is classified into 2 subtypes: DGAT1 (NM_010046) and DGAT2 (NM_026384) in mammals. Both DGAT1 and DGAT2 are ubiquitously expressed, with the highest levels of expression found in tissues that are active in triacylglycerol synthesis in the intestines and liver [19,20]. In triacylglycerol synthesis pathway, DGAT1 is not essential but seems to be better drug target for care of obesity because mice deficient in DGAT2 die shortly after birth [21-24]. In our study, DGAT1 mRNA expression level from intestines were higher than those of liver tissue, and DGAT2 mRNA expression level in liver was higher than those in intestines, such a tissue specific trend is consistent with a prior report [17]. DGAT2 mRNA expression levels in liver tissue derived from EF-exposed mice was lower than that in the control mice, and DGAT1 mRNA expression did not show significant differences between groups in this study. Although such an inhibitory effect was limited to liver tissue, to our knowledge, this is the first study to indicate that the EF of sinusoidal 50 Hz can effect in DGAT2 mRNA expression and pathways related to the regulation of lipid metabolism.

Our previous study about the EF-induced suppressive effect on the lactate synthetic pathway in stressed rats [10] would support the possibilities of an EF effect on energy metabolism, and another former study had also found that even though antioxidant activity was not influenced, plasma level of lipid peroxide was reduced by EF in rats exposed to the oxidizing agent, 2,2′-azobis(2-aminopropane) dihydrochloride (AAPH) [13]. At present, we propose a hypothesis that EF does not have catabolic effects, but does have anabolic effects on the lipid metabolism. Plasma level of triacylglycerol and free fatty acids were not influenced by EF but total cholesterol and phospholipid levels were. Down regulation in DGAT2 mRNA is consistent with this hypothesis. Even though triacylglycerol is a product of the DGAT-mediated pathway, plasma levels of triacylglycerol in the EF exposed mice did not show significant changes compared to the control mice. Because serum triacylglycerol decreased in DGAT1 null mice not but in DGAT2 null mice [21], the lack of change in plasma triacylglycerol in EF exposed mice is consistent. In addition, DGAT2 mainly mediates hepatic triacylglycerol synthesis [17]. In order to investigate the reason why DGAT2 mRNA down regulation in liver does not accompany the decrease in plasma triacylglycerol and free fatty acid, a study should be conducted on the effect of EF in triacylglycerol on liver tissue. Furthermore, because there are 2 pathways for triacylglycerol synthesis—the monoacylglycerol pathway and glycerol phosphate pathway [25], further studies should be conducted to identify the specific pathway that is affected by EF exposure. Diacylglycerol used by DGAT potentially originates from the hydrolysis of phosphatidic acid via a de novo pathway (Kennedy pathway), or the esterification of 2-monoacylglycerol or from triacylglycerol or phospholipid hydrolysis, which is also known as the remodeling pathway [19,20,25]. We observed decreased plasma levels of both total cholesterol and phospholipid levels in EF-exposed C57BL strain mice compared to the controls. These results also, in part, demonstrate that exposure to a sinusoidal EF leads to the inhibition of lipid anabolic pathways. The decreases in DGAT2 mRNA expression levels may be a secondary effect.

It should be noted that EF exposure did not affect animal growth. Such the effect suggest that EF exposure does not adversely impact the normal growth of the mice, and then, EF exposure could be a candidate for use in non-pharmacological modalities to control the induction of the DGAT molecule because control of DGAT expression has been examined as a potential pharmacologic approach in the care or prevention of an obesity or diabetes [24].

## Conclusion

In conclusion, our findings showed that EF exposure has an inhibitory effect on phospholipid and cholesterol production via the downregulation of DGAT2 mRNA expression. To our knowledge, this study is the first to describe the effects of EF exposure on a particular gene, DGAT2, which will help in further understanding of the subject. The mechanisms for the effects of EF on lipid metabolism are not well understand yet, but could be associated to lipid metabolism. To define the relationship of EF, DGAT expression, and regulation of lipid metabolism further experiments are needed.

## Materials and methods

### Animals

Seven-week-old C57BL/6 J male mice were purchased from CLEA Japan. All the mice were housed in polycarbonate cages, and maintained in a specific pathogen-free environment with light-controlled (lights-on from 07:00 am to 7:00 pm), and air-conditioned rooms (temperature: 24 ± 1°C, humidity: 50 ± 10%). The animals had free access to standard laboratory chow (CE-2; CLEA) and water ad libitum except for the period during EF exposure. The animals used in this study were cared for and used under the Guiding Principles for the Care and Use of Research Animals promulgated by the Obihiro University of Agriculture and Veterinary Medicine, Obihiro, Japan.

### EF exposure system

**Figure 3:**
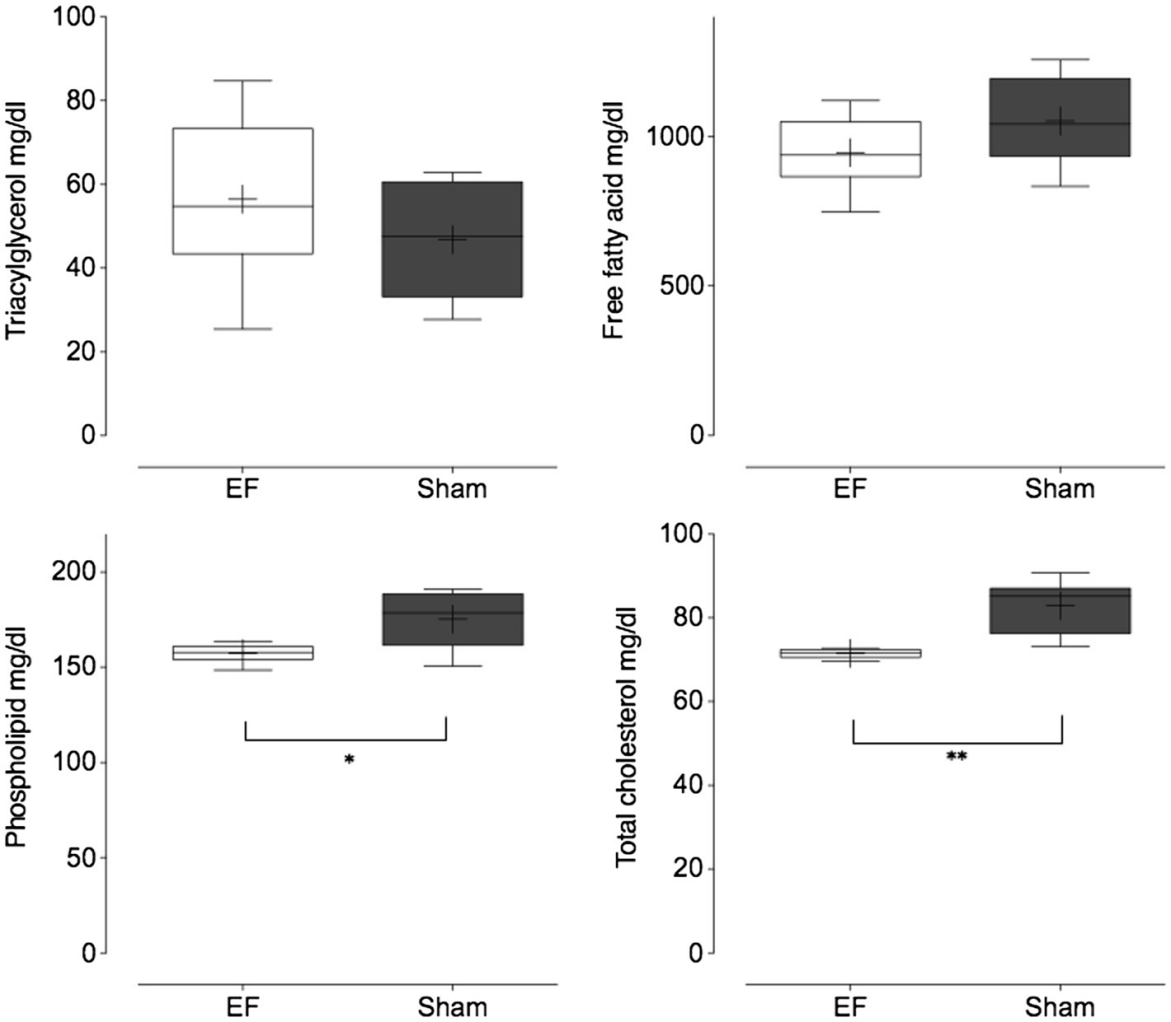
**A: The electric field exposure system. The electric field exposure system consists of electric exposure cages (also see B), a constant voltage unit, a high-voltage transformer unit, and a voltage controller**. B: The electric field exposure cage. A cylindrical plastic cage is placed between upper and lower stainless steel electrodes. The cylindrical cage has slits (length: 100 mm; width: 5 mm) at approximate intervals of 5 mm from each other to prevent smudges due to feces and saliva, which might disturb the formation of an electric field.

The EF exposure system was composed of 3 major parts, namely, a high voltage transformer unit (Healthtron; maximum output voltage: 9 kV; Hakuju, Japan), a constant voltage unit (Tokyo Seiden, Japan), and EF exposure cages [8-10,12,13]. The reason of usage a constant voltage unit is to avoid unexpected influences by electrical noises originated of commercial power supply (Tokyo Seiden, Japan). The exposure cage was comprised of a cylindrical plastic cage (diameter, 200 mm; height, 200 mm) and 2 stainless steel electrodes (1,000 × 600 mm) that were placed over and under the cylindrical cage (Figure 3A). The cylindrical cage has slits (length: 100 mm; width: 5 mm) around at intervals of 5 mm from each other (Figure 3B). The slits prevent smudges, due to feces and saliva, disturbing formation of stable EF. In order to establish the EF (50 Hz, 45 kV/m Root Mean Square) in the cage, a 50 Hz, 9 kV was applied to the upper electrode and lower one was connected to earth. An optical fiber voltmeter (FOVM 03; Sumitomo Electric, Japan) and a digital multimeter (Fluke 87 digital multimeter) were used to measure the field intensity and verify the system’s operation. The temperature within cylindrical cage did not change during EF exposure, and noise due to the discharge was not detected.

### Real-time quantitative polymerase chain reaction in DGAT

Eight-week-old C57BL/6 J mice were divided into 2 groups, EF and Sham groups (n = 6 per treatment group). Mice in the EF group were exposed to EF (50 Hz, 45 kV/m, 30 min/ day) for 11 days. The sham group mice were handled in a similar manner except that the EF condition was 0 V/m. Body weight was measured every 2 days. After the EF treatments on day 11, 500 μL of blood was collected under diethyl ether anesthesia, and the samples were centrifuged at 1500 × g for 10 min at 4°C and then stored at −80°C until used for blood property assays. All animals were euthanized after blood collection, and the livers, large and small intestines were collected and stored at −80°C for real-time quantitative polymerase chain reaction (real-time quantitative PCR). Total RNA was extracted from the tissues and purified according to a protocol provided by supplier (SIGMA, MO). Purified RNA was resuspended in sterile water. The cDNA was prepared using a TaqMan One Step RT PCR Master Mix Reagents kit (Applied Biosystems, CA). Real-time quantitative PCR was performed with specific doubly labeled probes in an ABI PRISM 7900 HT Sequence Detection System (Applied Biosystems, CA). The reaction mixture contained 10 μL of 2 × master mix without UNG (uracil-N-glycosylase), 0.5 μL of 40 × multiscribe and RNase inhibitor mix, 4 μL of 50 ng/μL RNA template, 1.1 μL RNase free double-distilled water, and 1 μL TaqMan gene expression assay probe. The reaction program was as follows: 48°C for 30 min, 95°C for 10 min, 45 cycles with 95°C for 15 s, and 50°C for 1 min. The data were analyzed by using SDS 2.1 software (Applied Biosystems). Mouse beta-actin, 18S rRNA, and glyceraldehyde-3-phosphate dehydrogenase (GAPDH) were used as internal control genes. Genorm software [26] was used for the selection of the most stable internal control gene. For a description of the primers and probes used for the internal control genes, see Table 1. Real-time quantitative PCR was conducted by each tissue from every individual mouse.

**Table 1:** Primer- or probe- sequences for real-time quantitative PCR

**Transcript**	**primer/Probe sequence (5′ to 3′)**	
DGAT	F	TGTCTGAGCGAACAAAGAATCTTG
	R	CGTGGGCGAACGGCTACT
		FAM-ACGATGGCACCTCAGA-MGB
DGAT2	F	GTCTTGGAGGGCTGAGAGGAT
	R	AAGAATAAAGGATCTGCCCTGTCA
		FAM-CCAGTGCCCCATCG-MGB
Beta-Actin	F	GCTCTGGCTCCTAGACCAT
	R	GCCACCGATCCACACACAGT
		FAM-ATCAAGATCATTGCTCCTC-MGB
18SrRNA	F	CGGCTACCACATCCAAGGAA
	R	GCTGGAATTACCGCGGCT
		FAM-TGCTGGCACCAGACTTGCCCTC-MGB
GAPDH	F	CGTCGCGAAGGATACTCT
	R	GGCAAGCAGATTTCACTGTGAAG
		FAM-TCGTCAACGGCCACCG-MGB

### Blood chemical analysis

Triacylglycerol, free fatty acid, total cholesterol and phospholipid in plasma samples were measured using an instrument TBA-120FR (Toshiba Medical Systems, Japan).

### Statistical analysis

Results were expressed as means ± SD. All statistical analyses were conducted using Prism 5.0d (GraphPad Software, CA). In body weight, two way repeated measures ANOVA and Bonferroni multiple comparisons were applied to compare the EF group with the Sham group. In mRNA expression analysis, ANOVA and Bonferroni multiple comparisons were conducted, and a t-test was conducted in the analyses of blood properties. Statistical significance of P < 0.05 was determined.

## Competing interests

The author(s) declare that they have no competing interests.

## Authors’ contributions

TH participated in the instrument setup, collection, analysis of data and writing of the manuscript; SH participated in the design, analysis and interpretation of data, performed the statistical analysis and writing of the manuscript; SMH and YYU participated in the instrument setup and collection; NI participated in the design; HS participated in the design, analysis and interpretation of data. All authors read and approved the final manuscript.
